# Multi-Cohort Transcriptomic Profiling of Medical Gas Plasma-Treated Cancers Reveals the Role of Immunogenic Cell Death

**DOI:** 10.3390/cancers16122186

**Published:** 2024-06-10

**Authors:** Antonios Gkantaras, Charalampos Kotzamanidis, Konstantinos Kyriakidis, Evangelia Farmaki, Kali Makedou, Georgios Tzimagiorgis, Sander Bekeschus, Andigoni Malousi

**Affiliations:** 1 Laboratory of Biological Chemistry, Medical School, Aristotle University, 54124 Thessaloniki, Greece; agkantar@auth.gr (A.G.); kmakedou@auth.gr (K.M.); tzimagio@auth.gr (G.T.); 2 Pediatric Immunology and Rheumatology Referral Center, 1st Department of Pediatrics, Aristotle University, 54124 Thessaloniki, Greece; farmakg@auth.gr; 3 Veterinary Research Institute, Thermi, 57001 Thessaloniki, Greece; kotzam@vri.gr; 4 UC Santa Cruz Genomics Institute, Santa Cruz, CA 95060, USA; kkyriaki@ucsc.edu; 5 ZIK Plasmatis, Leibniz Institute for Plasma Science and Technology (INP), 17489 Greifswald, Germany; sander.bekeschus@gmail.com; 6 Clinic and Policlinic for Dermatology and Venerology, Rostock University Medical Center, 18057 Rostock, Germany

**Keywords:** cold physical plasma, oncology, ICD, cold atmospheric plasma, meta-analysis, gene expression, transcriptomic profiling, Toll-like receptor signaling, oxidative stress

## Abstract

**Simple Summary:**

Medical gas plasma is a new modality in cancer treatment showing favorable results in preclinical and clinical trials; however, the cascade of molecular events underlying selective killing of cancer cells has not been fully elucidated. This study examines the hypothesis that cell death induced by gas plasma is mediated by a universal molecular mechanism. Using high-throughput data from eight transcriptomic studies on patient-derived prostate cultures, melanoma, breast, lymphoma, and lung cancer cells, we implemented two computational methodologies for re-analysis of single cohorts as well as meta-analysis of multi-cohort gene expression data. The results provide converging insights into the induction of immunogenic cell death in human tumors, reinforcing the potential of plasma treatment as an emerging immunotherapeutic approach in oncology.

**Abstract:**

The therapeutic potential of cold physical gas plasma operated at atmospheric pressure in oncology has been thoroughly demonstrated in numerous preclinical studies. The cytotoxic effect on malignant cells has been attributed mainly to biologically active plasma-generated compounds, namely, reactive oxygen and nitrogen species. The intracellular accumulation of reactive oxygen and nitrogen species interferes strongly with the antioxidant defense system of malignant cells, activating multiple signaling cascades and inevitably leading to oxidative stress-induced cell death. This study aims to determine whether plasma-induced cancer cell death operates through a universal molecular mechanism that is independent of the cancer cell type. Using whole transcriptome data, we sought to investigate the activation mechanism of plasma-treated samples in patient-derived prostate cell cultures, melanoma, breast, lymphoma, and lung cancer cells. The results from the standardized single-cohort gene expression analysis and parallel multi-cohort meta-analysis strongly indicate that plasma treatment globally induces cancer cell death through immune-mediated mechanisms, such as interleukin signaling, Toll-like receptor cascades, and MyD88 activation leading to pro-inflammatory cytokine release and tumor antigen presentation.

## 1. Introduction

Cold atmospheric plasma—hereinafter referred to as plasma—is a partially ionized gas at approximately room temperature, which comprises a unique source of reactive species, ultraviolet radiation, and electromagnetic fields [1,2]. The non-thermal nature, as well as its generation in atmospheric pressure, has enabled the direct application of plasma on biological tissues, laying the groundwork for potential medical applications [1,3,4]. Research over the last fifteen years has provided evolving evidence of the therapeutic potency of medical gas plasma in diverse biomedical fields, such as infectious diseases, wound healing and other dermatological disorders, dental and oral medicine, regenerative medicine, and tissue engineering, as well as oncology [2,3,5].

Oncological applications of plasma are of particular interest, as they have demonstrated substantial potential in inducing cancer cell apoptosis, inhibiting tumor growth, and enhancing the effectiveness of existing cancer therapies [6,7,8]. Recent studies revealed that plasma can selectively target malignant cells while sparing normal tissues, potentially reducing the side effects associated with conventional oncological treatments [9,10,11,12]. The observed cytotoxicity of plasma-treated malignant cell lines, in combination with the hitherto absence of reports of resistance to plasma treatment, has attracted attention for its promising therapeutic role in tumors [11,13]. The cost-effective manufacturing process of plasma-generating devices is a key factor driving the development of plasma medicine for cancer treatment, especially considering the high cost of targeted anti-cancer drugs [2,12].

The cytotoxic effect of plasma on malignant cells has been associated with its biologically active chemical compounds, namely reactive oxygen and nitrogen species (RONS) [13,14]. One hypothesis is that the often-lower cholesterol content of tumor cell membranes, which assists RONS-induced membrane lipid peroxidation and the subsequent large pore formation, in combination with the elevated expression levels of aquaporins, enable a massive influx of plasma-produced RONS into the cytosol of malignant cells compared to their non-malignant counterparts [11,15]. The accumulation of RONS inside cells can significantly disrupt the malignant cells’ antioxidant defense system, activating multiple signaling cascades and leading to growth arrest or cell death due to oxidative stress [14]. The most widely known biological pathways involved in ROS-induced cell death after plasma treatment include [16] nuclear factor erythroid 2-related factor 2 (Nrf2), extracellular regulated protein kinases (ERK) 1/2, c-Jun N-terminal kinase (JNK), mitogen-activated protein kinase (MAPK), and p53 signaling pathways [2,12,17,18,19,20,21,22,23].

Contrary to the numerous low-throughput approaches, there are limited systems biology methods to unravel the molecular mechanisms of plasma treatment efficacy against tumors through whole transcriptomic profiling in different cell cultures. In contrast, most research efforts have focused on single signaling pathways, investigating the plasma-induced dysregulation of a priori defined sets of genes, primarily through the combination of reverse transcription-quantitative polymerase chain reaction (RT-qPCR) and Western blot [22,24]. In addition, existing evidence is not conclusive as regards the potential synergistic antitumor effect of non-RONS constituents of plasma, such as freely charged particles, ultraviolet (UV) radiation, and electromagnetic fields, which in recent studies have been attributed significant inhibitory and apoptotic effects on diffuse histiocytic lymphoma and melanoma cells [13,25,26]. From a technical standpoint, definitive conclusions regarding the exact activation mechanisms of plasma as an anti-cancer agent remain further elusive due to the utilization of heterogeneous plasma-generating devices and different carrier gases and experimental protocol setting, as well as the evaluation of distinct outcomes of plasma treatment—either direct or indirect—in disparate malignant cell lines [27,28].

To address these issues, the present study sought to examine if cell death induced by plasma is mediated by a universal molecular mechanism in human cancer cells or if the biological effects of plasma treatment are unique to certain types of cancer cells, affecting specific signaling pathways. To this end, we performed a re-analysis and meta-analysis of whole transcriptomic profiles derived from plasma-treated human cancer cells that were investigated in pre- vs. post-treatment settings. We hypothesized that transcriptional aberrations are reproducible for certain dysregulated genes that are associated with common molecular activation mechanisms, leveraging the biological and technical heterogeneities of the cancer cells and experimental protocols. A particular emphasis was put on immunological phenomena, which are frequently observed in tumor animal models in the field of plasma medicine [29].

## 2. Materials and Methods

### 2.1. Search Strategy and Eligibility Criteria

A systematic search of published high-throughput gene expression studies was conducted in Pubmed from June 2022 to December 2023 and formulated following the PRISMA Statement guidelines [30]. All publications were written in English and combined human cancer cell lines with plasma treatment using the following search terms: (i) “Cold Atmospheric Plasma” AND Gene Expression, (ii) “Plasma Gases” [MeSH] AND “Gene Expression” AND “Cancer”, and (iii) (“Cold Atmospheric Plasma” OR “Non Thermal Plasma” OR “Plasma Gases” [MeSH] OR “Plasma Gases”/“therapeutic use*” [MeSH]) AND (“Gene Expression” OR “mRNA” OR “Transcriptomics” OR “Transcriptome” OR “Microarray” OR “RNA-seq”) AND Cancer. MeSH terminology was set to broaden the searches and encompass related concepts, coping with the challenge of gas plasma synonyms. Eligible studies meet all the following inclusion criteria: (i) Original work evaluating gene expression patterns in human cancer cell lines or tissues after plasma treatment, (ii) gene expression profiling performed by high-throughput methods, either microarrays or RNA sequencing (RNA-Seq), (iii) transcriptomic data were publicly available through Gene Expression Omnibus (GEO) [31], (iv) transcriptome analysis was focused on coding RNAs, and (v) the datasets included both untreated and plasma-treated cancer samples.

The eligible studies were mapped to the corresponding accessions of the GEO data series and checked for duplicated records. Each GEO accession was further examined for eligibility by carefully reviewing the information provided on the GEO Accession viewer platform. Furthermore, an independent search was conducted for gene expression data series in GEO using the keywords “Cold Atmospheric Plasma” and “Non-Thermal Plasma”. Additional query terms such as “medical gas plasma”, “low-temperature plasma”, and “cold physical plasma” were also used. Additional filters were applied to narrow down suitable datasets as follows: “Series” (entry type) and “Homo sapiens” (organism). Data series not containing human cancer samples or expression profiling by array or high throughput sequencing data were excluded. All the non-redundant studies identified through gene expression data repositories or medical literature databases built the final set of eligible datasets.

### 2.2. Single-Cohort Data Collection and Re-Analysis

The analytical scheme applied to eligible studies included data acquisition, sample filtering and normalization, differential gene expression analysis, pathway enrichment analysis, and ancestor pathway comparison. Each dataset was re-analyzed separately using the same parameters, except for the statistical significance level. All bioinformatics analyses were performed in R v4.1.3 and supported by Bioconductor packages [32]. Gene expression matrices of each data series were fetched using GEOquery 2.72.0 [33] package, together with the sample information and platform annotation. All individual datasets were quantile normalized and log_2_-transformed according to the data processing protocol of each study. The mean expression level was assigned to each target gene in case of multiple probe values. Exploratory analysis was performed using Principal Component Analysis (PCA), following quality assessment/filtering and differential expression analysis by the limma 3.60.2 package [34]. The cutoff for the differentially expressed genes (DEGs) was set by the adjusted *p*-value ≤ 0.05 and |log_2_FC| ≥ 1 (Table S1). Less stringent thresholds (|log_2_FC| ≥ 1, *p*-value ≤ 0.05) were set in case either no statistical significance was attained, or statistical analysis could not be performed due to a small sample size (one sample per category). DEGs were exported for each study and comparatively analyzed to map shared deregulated genes with plasma-induced mechanisms using in-house R scripts, bioDBnet 2.1 [35], and the BiomaRt 2.60.0 [36] package.

### 2.3. Multi-Cohort Meta-Analysis

Multi-cohort transcriptomic meta-analyses were implemented by MetaIntegrator 2.1.3 [37]. SOFT files were downloaded for all experiments including expression and phenotypic annotations. Platform-specific probe IDs were mapped to gene names according to the GPL annotation files and quality-checked for log_2_-transformed and quantile normalized values, as well as minimum sample size. Effect sizes (ES) were calculated as Hedges’ adjusted g and summarized using the random effects inverse variance model, where each effect size was weighted based on the intra-dataset and inter-dataset variances of each gene, estimated by the DerSimonian–Laird method. The effect size heterogeneity across studies was assessed by Cochrane’s Q value, and the statistical significance of the heterogeneity level per gene was estimated by the *p*-value of Cochrane’s Q. Fisher’s sum of logs was calculated for the up-regulated and down-regulated genes to combine *p*-values across datasets. *p*-values under a chi-squared distribution were calculated for each gene, and Benjamini–Hochberg false discovery rate (FDR) correction was performed across all genes. Meta-analysis was performed on five studies that matched the minimum sample threshold. Genes matching the |ES| ≥ 1 and the combined Fisher’s FDR ≤ 0.05 in at least five studies were finally selected. The contribution of individual studies for each up-regulated gene was visualized using forest plots.

### 2.4. Comparative Functional Enrichment Analysis

To illustrate the underlying molecular mechanisms associated with plasma treatment efficacy in individual datasets, we applied pathway enrichment analysis using ReactomePA 1.48.0 [38]. In addition, pathfindR [39] was applied to search for active subnetworks using protein–protein interaction networks of BioGRID 3.2 [40]. The enriched pathway terms were subsequently retrieved using the identified subnetworks. rbioapi 0.8.1 [41] was used to retrieve the hierarchical structure of each pathway and the top-level pathway ancestors through the REACTOME API services. A hypergeometric model implemented gene over-representation analysis of meta-analysis results against Reactome pathways.

## 3. Results

### 3.1. Summary of Eligible Cohort Studies

**Figure 1:**
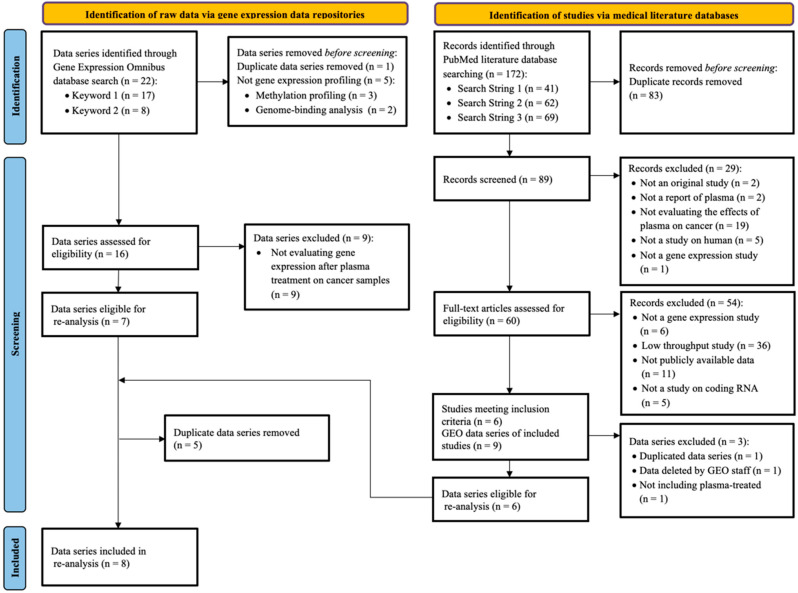
Flow diagram of the search strategy and gene expression dataset selection process.

Systematic literature review and public datasets search resulted in 172 and 22 studies, respectively. Eight cohorts fulfilled the inclusion criteria (Figure 1). Then, 78% of the eligible studies were excluded as they did not perform pre- and post-treatment profiling, they did not provide the raw gene expression data, or they did not perform whole transcriptome analysis. One study was missed in the search for matching repository records, while two cohorts meeting the inclusion criteria were missed by the literature review. Among those excluded, 11 RNA-seq and microarray cohorts did not provide raw transcriptome data, and one performed profiling of non-coding RNAs. The design details of the selected studies are summarized in Table 1, including sample information, the plasma treatment device/carrier gas, and the high-throughput platform that performed gene expression profiling.

As expected, the eligible cohorts exhibit wide experimental heterogeneity regarding the plasma production method, application mode, and exposure time. Six studies used a dielectric barrier discharge (DBD) device to produce plasma. Argon—alone or in combination—was the carrier gas used in six studies, and helium was used in the remaining two studies. Five transcriptionally distinct human cancer lines/cancer types were included, namely A549 lung carcinoma epithelial cells, U937 histiocytic lymphoma cell line, SK-MEL-147 melanoma cell line, MCF-7 breast cancer cells, and prostate basal epithelial cultures generated from patient-derived cancer tissue. MCF-7 cells were analyzed in four studies, including two MCF-7 modified cell lines that were resistant to Tamoxifen and Paclitaxel. The studies were performed in whole transcriptome microarray platforms targeting 47,319–58,201 probes.

Each study was originally designed to address different research questions and produced cell-specific findings on the effect of plasma treatment. For example, plasma treatment of SK-MEL-47 melanoma cells was associated with an inhibition of migration and disorganization of the actin cytoskeleton that was mediated through multiple signaling pathways. The analysis of the lymphoma U937 cells investigated the induction of apoptosis by plasma. The major effect of the medical gas plasma treatment over the A549 cells was the activation of MAPK and p53 signaling pathways and the modulation of genes related to cellular proliferation and differentiation. The induction of oxidative stress and the activation of NOTCH signaling were the main findings of the analysis of the patient-derived prostate cancer cultures. The analysis of the Paclitaxel-resistant and Tamoxifen-resistant MCF-7 revealed that plasma inhibited the growth of cancer cells and recovered drug sensitivity. Direct plasma treatment was applied in all except the SK-MEL-147 study, in which indirect treatment was preferred to avoid mechanical stress to the cells due to the gas flow.

### 3.2. Single-Cohort Transcriptomic Re-Analysis Revealed Consistently Up-Regulated Genes

**Figure 2:**
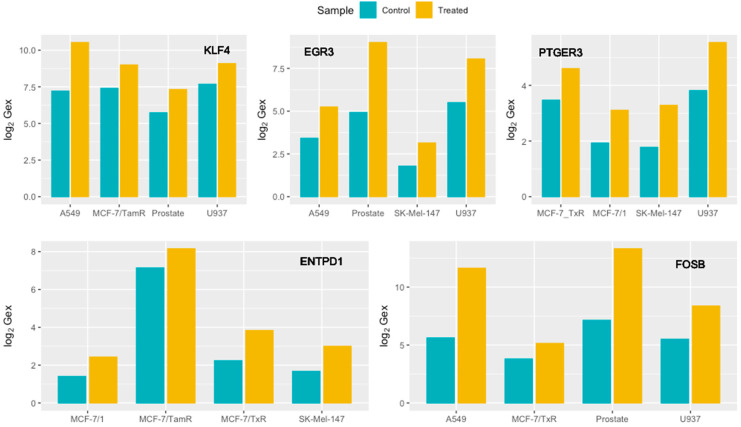
log_2_-scaled, quantile normalized expression levels (log_2_Gex) of the statistically significant genes that were found to be up-regulated in the plasma-treated samples of four cohorts.

The sample size for each cohort was defined following manual curation of the metadata in order to standardize the treatment times across studies (Table S1). The resulting 30 pre- and post-treatment samples were exposed for 1–3 min to plasma except for MCF-7, which was treated ten times for 30 s with 1 h intervals [45]. Sample collection after exposure ranged on a scale of a few hours. In the exploratory analysis, the cohorts that included at least two samples per class showed increased explained variability ranging between 63.9 and 88.7% for the first two principal components (Figure S1). Hierarchical clustering of these studies revealed significant gene deregulation in the pre- and post-treatment setting across most studies, except for the Paclitaxel-resistant MCF-7 cell line. The standardized re-analysis of individual cohorts identified 1074 deregulated probes on average, including uncharacterized gene loci (LOC, XLOC) and non-coding RNAs, as well as multi-to-single probes-gene mappings. Quantitative analysis of deregulated genes across cohorts presents no clear pattern or trend between plasma-treated and control samples, while no significant correlation between the sample size and the number of deregulated genes was observed. Nineteen genes were found to be significantly deregulated in at least four studies, most of them with mixed responses across cohorts (Table S2). MCF-7/1 cell line, the patient-derived epithelial prostate cells, and the SK-MEL-147 melanoma cells are the most representative cohorts, as they include most of the commonly deregulated genes. Among these, *Fosb, Entpd1, Ptger3, Egr3*, and *Klf4* were found to significantly increase their expression levels in four cohorts (Figure 2).

### 3.3. Common Biological Aberrations Caused by Plasma-Treated Cancer Cells

**Figure 3:**
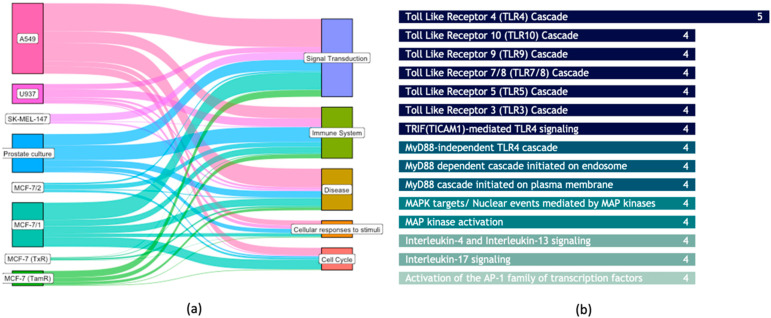
(**a**) Flow visualization of linking plasma-treated cancer cells and pathway classes. (**b**) Immune response pathways involved in plasma treatment and the number of cohorts, out of eight, showed statistically significant deregulation.

Single-cohort analysis revealed significant overlapping of the deregulated molecular pathways. By retrieving the event hierarchy, we aggregated pathways into the top-level ancestors and comparatively evaluated the primary sources of deregulation to pinpoint distributional differences across different cancer types. The main pairwise connections between cancer cells and deregulated pathways are shown in Figure 3a. As expected, pathways activating signal transduction are commonly deregulated in all cohorts and cover a broad spectrum of descending signaling events (138 unique sub-pathways), such as receptor tyrosine kinase signaling, (NRTKs, SCF-KIT), NOTCH signaling pathway, MAP kinase activation, and nuclear events mediated by MAP kinase pathways, and ESR-mediated signaling.

Immune pathways involve mainly the innate system and cytokine signaling and not adaptive immunity. Commonly deregulated pathways were within 91 district pathways in the immune system hierarchical events (Figure 3b). Toll-like receptor 4 (TLR4) cascade is the most prevalent pathway. TLR4 is known to trigger cascades of reaction that lead to the local secretion of pro-inflammatory cytokines, including IL-1 and IL-6 [47]. MyD88-independent TLR4 cascade is a sub-pathway that is significantly deregulated among other TLR4 cascades, known to recruit TRIF to the TLR complex that stimulates pathways leading to the production of pro-inflammatory cytokines and induces cell death. Other TLR cascades are also significantly deregulated, implying a global plasma-medicated activation of toll-like receptors. The anti-tumoral role of TLR agonists has shown to be effective in the treatment of several cancers in several ways, e.g., the recruitment of leukocytes, particularly cytotoxic T, helper T, and NK cells. TLR5 and TLR10 deregulation mainly involve the MyD88 cascade initiated on the plasma membrane, leading to the recruitment of the IL1 receptor associated with the kinase family IRAK. MyD88-dependent cascade initiated on endosome is the dominant sub-pathway deregulated among TLR7/8 and TLR9 cascades. Deregulation of cytokine signaling mainly involves interleukins 4, 13, and 17, which play pivotal roles in host immune defenses in response to various stimuli by activating several downstream signaling pathways. The activation of AP-1 transcription factors is a descendant pathway of the innate immune system and cytokine signaling and is involved in MAP kinase activation and nuclear events mediated by MAP kinases.

Despite the existence of common deregulated immune signaling pathways among the different plasma-treated cancer cell lines (TLR cascades, MyD88 signaling, IL-4/-13/-17 signaling, MAPK activation, and related up- and downstream pathways), our analysis demonstrated that specific cancer cell lines displayed distinct activation patterns of immune-related pathways after plasma treatment. As illustrated in Table S3, SK-MEL-147 plasma-treated cells displayed less overall activation of immune-related pathways (*n* = 2; FLT3 signaling and negative regulation of FLT3), whereas, in plasma-treated prostate cancer cells, there is an aberrant up-regulation of NFkB activation pathways (*n* = 5) which is not evident in the other studied cancer cell lines. The complete list of the unique immune-related pathways activated after plasma treatment in a single cancer cell line is presented in Table S4.

Disease pathways refer mainly to diseases of signal transduction by growth factor receptors, e.g., PI3K/AKT and ERBB2 signaling. Pathways related to cellular responses to stimuli are also commonly deregulated in plasma-treated cells. These include oxidative stress-induced senescence, heat shock factor 1 (HSF1) transcription factor activation, and senescence-associated secretory phenotype (SASP). Finally, cell cycle deregulation involves mainly the G1 phase of the mitotic interphase, the G1/S transition, as well as the G2 and G2/M transition phases. Despite the convergent biological effects towards signal transduction and immune system, plasma treatment revealed variations in the distribution of the pathways across cancer cells. The differentially expressed genes identified in A549 and SK-MEL-147 plasma-treated cells are mainly associated with signal transduction processes, while U937 and patient-derived prostate cells mainly affect pathways of the immune system. The evidence is not conclusive for the MCF-7 treated cells. Considering the small sample sizes and the experimental heterogeneities, the comparison across cancer cells should be further elaborated to draw safe conclusions.

### 3.4. Multi-Cohort Transcriptomic Analysis

**Figure 4:**
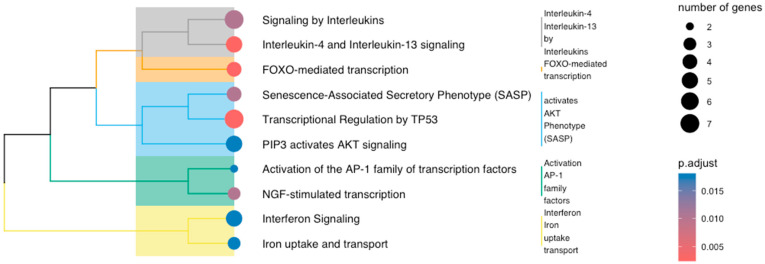
Pathways affected by the up-regulated genes of the meta-analysis with FDR ≤ 0.05.

A signature of 34 positively regulated genes was found in all plasma-treated samples with Fisher’s FDR < 0.05 (Table S5). The geometric mean of these genes was also increased in the plasma-treated samples in two of the three datasets that were not eligible for meta-analysis. The forest plots in Figure S2 represent the log_2_ z-scaled Hedges’ g of the most significantly up-regulated genes (Fisher’s FDR < 0.01). *FosB, HSPA1B, JUN*, and *KLF4* were also found to be among the deregulated genes in the re-analysis of individual cohorts. In addition, the meta-analysis revealed the presence of oxidative stress-associated transcription factors *FOS, EGR1*, and *NR4A1*, which have been reported to be up-regulated in intestinal stem cell-derived organoids under plasma treatment [48]. Both *NR4A1* and *NR4A2* were detected in the meta-analysis with effect size > 1.5, while no such evidence was reported in the re-analysis of individual reports. Cytokine signaling, mainly interferon and signaling by interleukins, as well as processes of the innate immune system, e.g., activation of the AP-1 transcription factors, are the main common deregulated pathways in the combined five-cohort meta-analysis (Figure 4).

**Figure 5:**
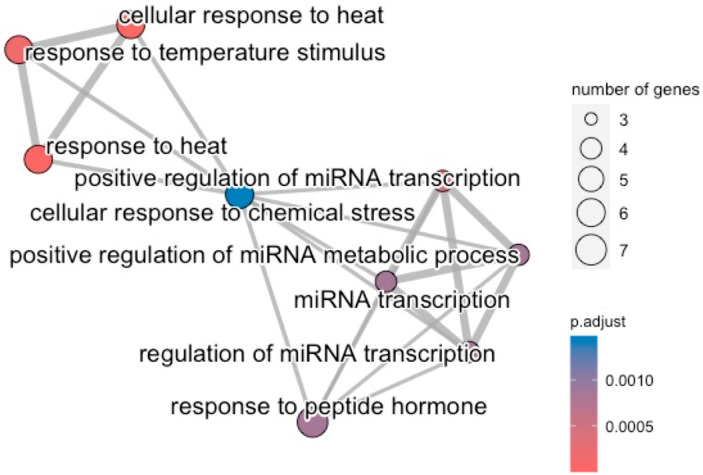
GO biological processes that are affected by the up-regulated genes.

Plasma-mediated deregulation also involves generic transcription pathways, including regulation by TP53 and FOXO-mediated transcriptional events. Cellular responses to stress, such as the senescence messaging secretome, validate the outcome of the single-cohort analysis and further verify the involvement of inflammatory and immune-modulatory cytokines. The broader biological roles of the up-regulated genes mainly involve processes related to response to stimuli, e.g., heat, or stress factors, e.g., chemical stressors. A statistically significant association between the up-regulated gene set and processes associated with the regulation of miRNA transcription, mainly positive ones, is also observed (Figure 5).

## 4. Discussion

Systematic research on plasma-induced effects during the last decade has revealed the main sources of molecular aberrations. However, no integrated analysis has determined the genes and pathways that are consistently deregulated across different cancer types. To increase generalizability and in-depth investigation, we applied two computational approaches: re-analysis of individual cohorts and multi-cohort meta-analysis. Re-analysis enables thorough contextual understanding and control over the analysis parameters and confounding factors, while the meta-analysis increases the sample size, leading to higher statistical power, addressing heterogeneities, and offering better generalization performance.

The single-cohort analysis revealed five genes that significantly increase the expression level. Fosb is an oxidative stress-associated transcription factor that is known to be up-regulated in global stress response induced by plasma treatment [48]. Klf4 is involved in diverse cellular processes, including proliferation, differentiation, and control of gene transcription, and has been experimentally verified to play a positive role in the regulation of nitric oxide biosynthetic process [49]. Entpd1 is an enzyme expressed on the surface of various cell types, including immune cells and endothelial cells. Its activity has been implicated in modulating purinergic signaling, which plays a role in inflammation, immune responses, and other cellular processes. Ptger3 is involved in diverse functions, including vasoconstriction, inhibition of adenylate cyclase, and modulation of immune responses. Ptger3 is also known to inhibit tumor cell growth [50] and was found to be significantly up-regulated in plasma-treated cells [25]. Egr3 is a transcription factor that plays a role in the regulation of gene expression, particularly in response to various stimuli and stressors. Early growth response genes are expressed in many different cell types, and their expression is rapidly induced in response to apoptotic signals and tissue injury, while it has been shown that cell death induced by plasma was enhanced by Egr1 overexpression [51]. The meta-analysis revealed two additional genes of the nuclear receptor family, namely, *NR4A1* and *NR4A2*. Interestingly, *NR4A1* and *NR4A2* code for proteins that act as nuclear transcription factors. The Nr4a subfamily of nuclear receptors is rapidly induced in response to stimuli, such as oxidized lipoproteins, and plays a significant role in the regulation of innate and adaptive immune responses [48]. The nuclear receptors NR4A1/2 have a dynamic role in both CD4+ and CD8+ T-cell responses, regulating the balance between immune activation and exhaustion, as well as affecting the production of cytokines [52]. The pivotal role of NR4As in the exhaustion of cytotoxic CD8+ T-cells suggests that adjusting their expression through plasma treatment represents a promising therapeutic strategy to enhance anti-cancer immune responses. Recent research reported that the development of NR4A inducers and NR4A1/NR4A2 antagonists for hematologic malignancies and solid tumors, respectively, may have significant therapeutic implications [53,54]. The regulation of *NR4A1/2* gene expression through plasma treatment shown in this study could be a promising, low-cost therapeutic strategy in a wide range of malignancies, also enhancing immune surveillance [55,56]. This is an interesting observation; however, further evidence is needed to explore the potential causal association of these genes under plasma treatment.

The main finding confirmed that RONS-induced cell death is one of the major mechanisms of plasma treatment efficacy, as indicated by the enrichment of molecular pathways associated with oxidative stress. In response to the intracellular accumulation of RONS, the up-regulation and activation of ERBB2 tyrosine kinase receptors induces the subsequent downstream activation of MAPK cascades and PI3K/AKT signaling, promoting apoptosis [57]. In addition, Rho GTPases and signaling proteins involved in Insulin/IGF1R signaling contain redox-sensitive cysteine or tyrosine residues, which act as rapid sensors of cellular redox imbalance and regulate gene expression accordingly [58,59]. Several studies have also demonstrated the crosstalk between Notch signaling and oxidative stress, with the transcription factor Nrf2 playing a pivotal role in both the transcriptional regulation of Notch receptors and the orchestration of antioxidant responses [60]. It has to be highlighted that Nrf2 signaling has been acknowledged as the major molecular mechanism of the efficacy of plasma in chronic wound healing [13,44]. Nevertheless, studies implicating Nrf2 signaling as an active regulatory transcriptional network after plasma treatment in cancer cell lines are limited [21,42]. Even though the majority of these signaling pathways have been reported as isolated findings in previous studies, our study underlines their concurrent activation in response to plasma treatment, providing a comprehensive overview of the RONS-associated molecular pathways triggered by plasma in multiple human cancer cell lines.

Recently, it has been proposed that plasma treatment can induce cancer cell death through immune-mediated mechanisms. The existing literature has focused mainly on the direct effects of plasma on immune cells, demonstrating enhanced antitumor capacity of macrophages, more effective tumor antigen presentation, and augmented activity of cytotoxic CD8+ T-cells following exposure to plasma [12,14]. In addition, the observed release of damage-associated molecular patterns (DAMPs) from plasma-treated cancer cells further suggests that plasma treatment can induce immunogenic cell death (ICD) [12,61,62].

To our knowledge, this study is the first to implicate specific dysregulated immune pathways associated with ICD in response to plasma treatment, such as TLR cascades. Specifically, the secretion of HMGB1 protein, which appears up-regulated in post-plasma treatment, induces TLR4 and downstream MyD88 activation, leading to pro-inflammatory cytokine release and robust tumor antigen presentation. Studies on murine models have demonstrated that both TLR4-deficient and MyD88-deficient mice display reduced capability to induce ICD following vaccination with autologous tumor cells, suggesting that the HMGB1/TLR4/MyD88 axis is indispensable for ICD [63,64]. Moreover, activation of the TLR3 by plasma-treated cancer cell-derived dsRNA can induce potent type I interferon responses via IRF3 signaling, as well as activation of the TRIM/caspase-8 pathway, resulting in immunogenic cancer cell apoptosis [63,65]. The reported association between the up-regulated genes and processes associated with the regulation of miRNA transcription provides insights for elucidating the role of the non-coding genome that needs further investigation [66].

Nevertheless, the degree of activation of immune signaling pathways varied among cancer cell lines. This might reflect the sensitivity of each cancer cell line to immunogenic cell death after plasma treatment, similarly to the sensitivity of malignant cells to plasma-induced oxidative cell death, which depends on the capability of each malignant cell type to cope with oxidative stress [28,67]. In addition, the distinct immune-related pathways enriched in each plasma-treated cancer cell line might provide potential targeted therapeutic implications in case of applications of plasma-assisted immunotherapy [28,68]. For example, the enrichment of multiple NFkB activation pathways in plasma-treated prostate cancer cells may denote that the therapeutic regulation of NFkB activation could possibly augment the therapeutic efficacy of plasma treatment in prostate cancer cases [69]. On the other hand, in cancer cell lines where plasma treatment achieved poor immune activation, further induction of the involved immune pathways, such as the use of an FLT3 agonist in the case of SK-MEL-147, would have a synergistic effect in inducing immunogenic cell death in cancer cells [70,71]. These hypotheses should be evaluated in in vitro studies to further examine the role of medical gas plasma in eliciting potential ICD-enhancers in distinct cancer types [72].

## 5. Conclusions

In this study, we sought to examine whether the transcriptomic alterations induced by plasma treatment in cancer cells are shared across different cancer types. Cancer cell lines, patient-derived cells, and cultured cells resistant to anti-cancer drugs were included in this study. The experimental heterogeneity of the cohorts including the diverse plasma sources, biological models, treatment duration, and microenvironmental conditions can impact the generalizability of their findings. In addition to the experimental heterogeneities, the results can also be affected by the computational methods, including the differential gene expression analysis algorithms and the statistical thresholds. The obvious effect is that it is hard to extrapolate the findings from one study to the other on gene level and therefore to identify common biomarkers linked to the response levels of gas plasma-treated cells. An important confounding factor is the small sample sizes of the cohorts that increase the risk of random variability and the impact of random fluctuations. In the absence of standardized protocols to deal with the variability experimental approaches, the present work focuses on mitigating the heterogeneity of the in silico analyses by merging the original data to increase statistical power (meta-analysis approach), and standardizing the normalization, filtering, and differential analyses (re-analysis approach). Both approaches verified the robustness of the findings on the pathway level, contrary to the evident variability on the gene level among cohorts. To increase their statistical power, individual cohorts could benefit by the use of biological replicates. In addition, conducting time-course transcriptomic profiling at multiple time points post-treatment can provide insights into the temporal dynamics of plasma-induced transcriptional changes and the robustness of the transcriptional analysis. This would be particularly beneficial in primary cancer tissue studies, in which the level of heterogeneity is significantly higher [28,67].

The results provide further insights into the induction of ICD in human tumors in response to plasma treatment, underpinning the potential of plasma as an emerging immunotherapeutic approach in oncology. By adopting a systems pharmacology approach, previously applied to develop machine learning models and to identify biomarkers of anti-cancer drug response [73,74], it would be feasible to predict plasma-induced cell death and develop personalized therapeutic applications based on the mutational, transcriptional, and copy number profiles of cancer cell lines and patient-derived samples.

## Figures and Tables

**Table 1:** Summary of the gene expression cohorts matching the eligibility criteria.

GEO ID	Samples	Cancer Cells	Cancer Type	Device	Carrier Gas	Treatment	Platform	Citation
GSE119052	12	Patient epithelial prostate	Prostate cancer	Dielectric Barrier Discharge	Helium (0.3% mol. oxygen)	Direct	Affymetrix Human Clariom D Assay	[42] *
GSE59997	15	A549	Lung cancer	Dielectric Barrier Discharge	Helium	Direct	Affymetrix Human Gene Expression Array	[18]
GSE76022	6	U937	Lymphoma	Plasma Jet	Argon ± Nitrogen	Direct	Affymetrix Human Genome U133 Plus 2.0	[43] *
GSE65972	32	SK-Mel-147	Melanoma	Plasma Jet kinpen09	Argon	Indirect	Agilent-062647 INP_039494_Human_GE_v2	[44] *
GSE95208	3	MCF-7 (+MCF-7/TamR)	Breast Ca (Tamoxifen-resistant)	Dielectric Barrier Discharge	Argon	Direct	Illumina HumanHT-12 V4.0 beadchip	[45]
GSE110117 ^	2	MCF-7/1	Breast cancer	Dielectric Barrier Discharge	Argon	Direct	Illumina HumanHT-12 V4.0 beadchip	[45] **
GSE117491 ^	2	MCF-7/2	Breast Ca	NA	NA	NA	Agilent-072363 SurePrint G3 Human GE v3	NA
GSE131480	7	MCF-7 (+MCF-7/TxR)	Breast Ca (Paclitaxel-resistant)	Dielectric Barrier Discharge	Argon	Direct	Agilent-072363 SurePrint G3 Human GE v3	[46]

NA = Νο available information. * Both cited also by Ji HW et al. [25] (original study) and used for bioinformatics re-analysis. ** Citation not reported in GEO. ^ Data series are part of the same GEO super series (GSE117683). The sample sizes represent the total number of transcriptomes included in each study.

## Data Availability

Data are contained within the article and Supplementary Materials.

## Supplementary Materials

The following supporting information can be downloaded at: https://www.mdpi.com/article/10.3390/cancers16122186/s1, Figure S1: PCA and cluster visualization of individual datasets. Genes with <10% of variance are not considered in the component analysis. Datasets including groups of one sample are not depicted. Figure S2: Forest plots of the multi-cohort analysis (Fisher’s FDR ≤ 0.01). The size of the blue boxes is proportional to the number of samples in the study, and light blue lines indicate the standard error of the effect sizes for each study (95% confidence interval). The summary effect size across studies is shown as orange diamonds. The remaining genes for which FDR ≤ 0.05 are: *FOS*, *RAE1*, *TNFRSF10D*, *DNAJB1*, *MIR22HG*, *CDKN1A*, *ATP6V0B*, *PTGS2*, *PLAUR*, *PRKCB*, *CSRNP1*, *EMP1*, *ZNF263*, *TIGD1*, *BCL6*, *SFN*, *HLA-E*, *RPN1*, *PTGES2*, *ZNF34*. Table S1: Number of samples, treatment time, and statistical significance thresholds for deregulated genes. Table S2: Name and description of genes that were found to be significantly deregulated in at least four studies. Table S3: Immune-related signaling pathways activated after plasma treatment and the corresponding studies. Table S4: List of uniquely activated immune-related pathways per study. Table S5: A signature of 34 positively regulated genes based on the meta-analysis of the cohort studies (Fisher’s FDR < 0.05).
