# Supplementary material for: Multi-Cohort Transcriptomic Profiling of Medical Gas Plasma-Treated Cancers Reveals the Role of Immunogenic Cell Death
Source: Cancers (Basel). 2024 Jun 10;16(12):2186. doi: 10.3390/cancers16122186 (PMC11201794; doi:10.3390/cancers16122186)
Supplement: Supplementary file 1 [file cancers-16-02186-s001.zip › Figures_S1_and_S2.pdf]

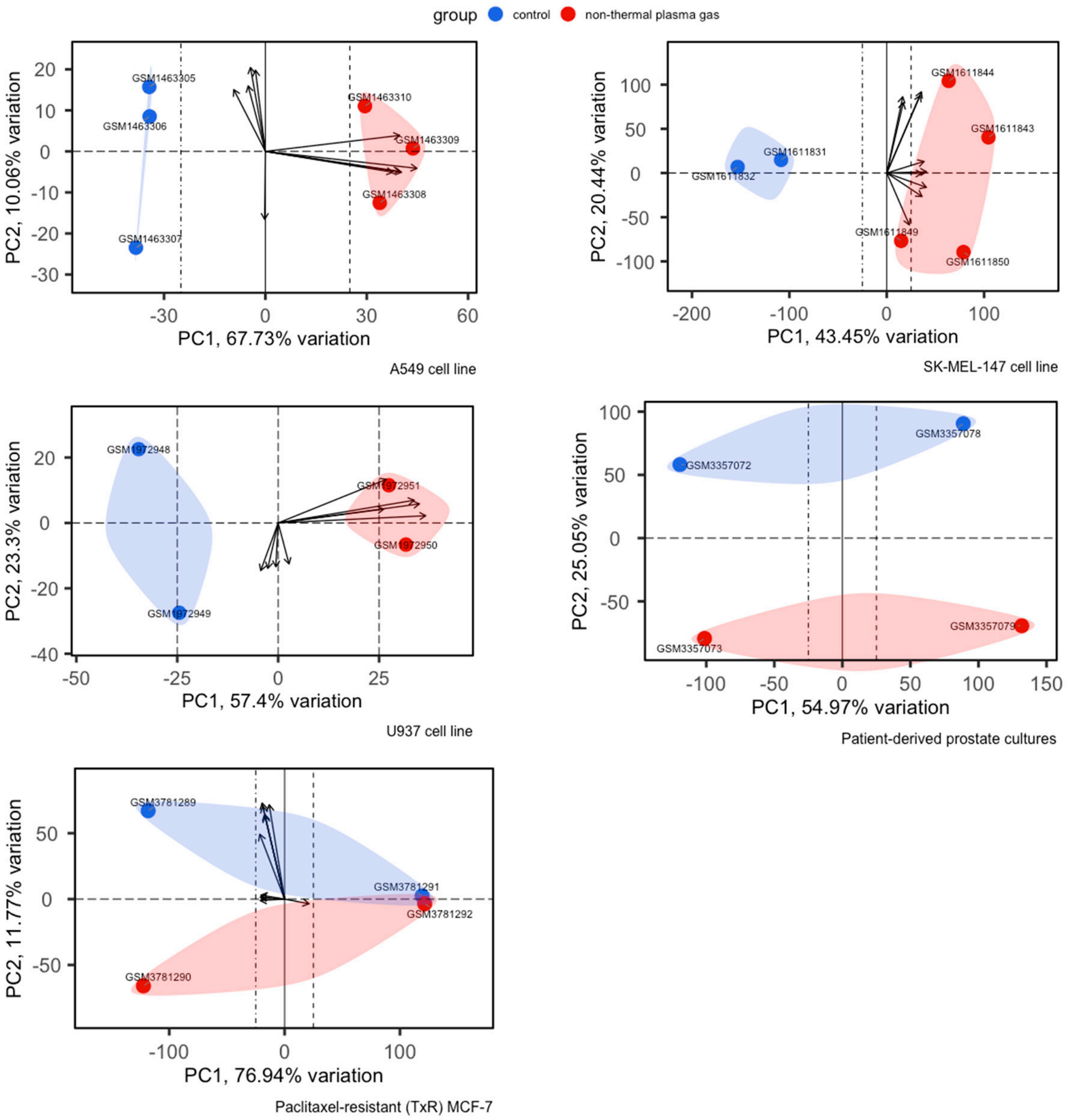

Figure S1. PCA and cluster visualization of individual datasets. Genes with <10% of variance are not considered in the component analysis. Datasets including groups of one sample are not depicted.

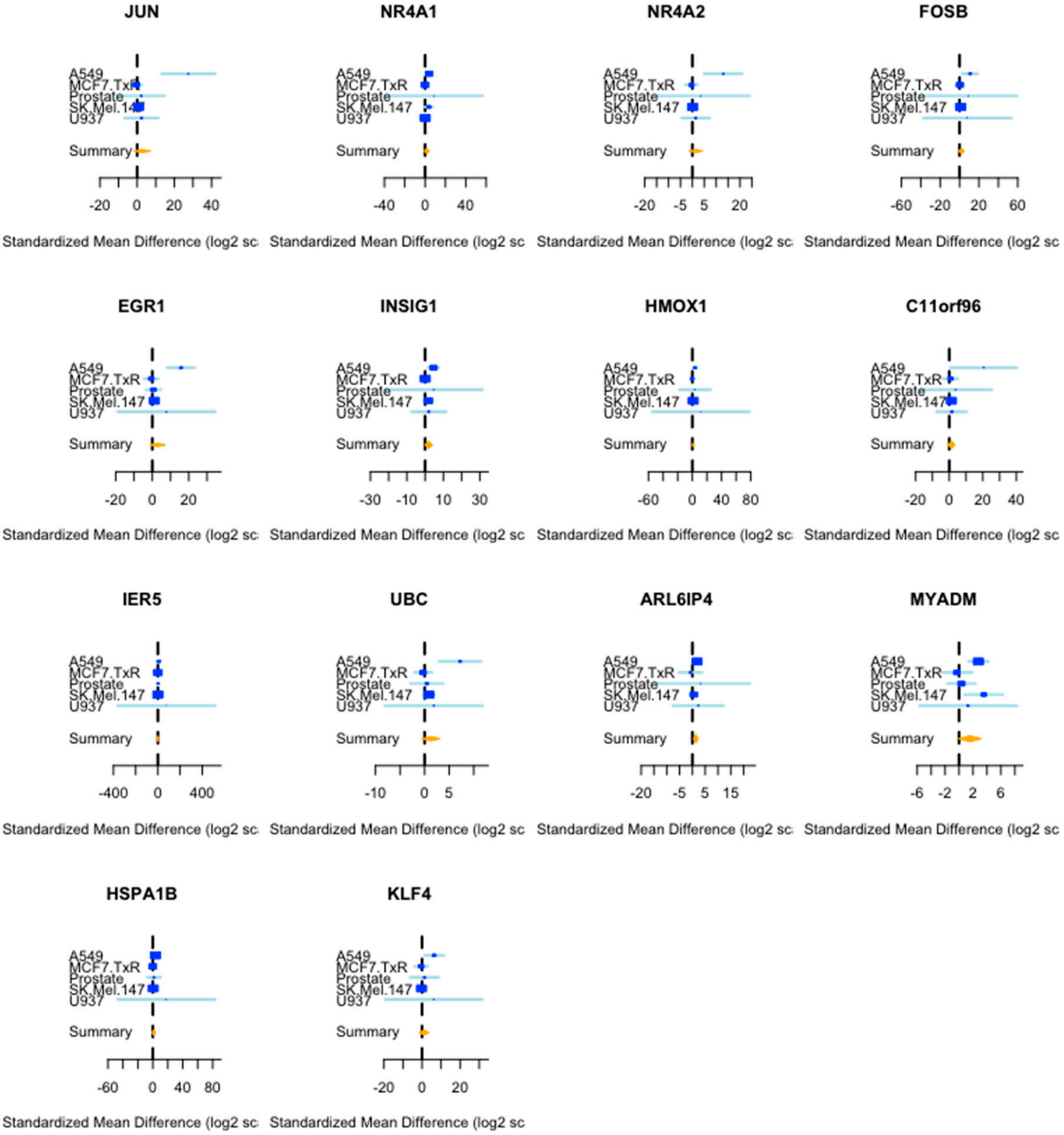

Figure S2. Forest plots of the multi-cohort analysis (Fisher's FDR <= 0.01). The size of the blue boxes is proportional to the number of samples in the study and light blue lines indicate the standard error of the effect sizes for each study (95% confidence interval). The summary effect size across studies is shown as orange diamonds. The remaining genes for which FDR <= 0.05 are: *FOS*, *RAE1*, *TNFRSF10D*, *DNAJB1*, *MIR22HG*, *CDKN1A*, *ATP6V0B*, *PTGS2*, *PLAUR*, *PRKCB*, *CSRNP1*, *EMP1*, *ZNF263*, *TIGD1*, *BCL6*, *SFN*, *HLA-E*, *RPN1*, *PTGES2*, *ZNF34*.
