# Supplementary material for: Multi-Cohort Transcriptomic Profiling of Medical Gas Plasma-Treated Cancers Reveals the Role of Immunogenic Cell Death
Source: Cancers (Basel). 2024 Jun 10;16(12):2186. doi: 10.3390/cancers16122186 (PMC11201794; doi:10.3390/cancers16122186)
Supplement: Supplementary file 1 [file cancers-16-02186-s001.zip › Tables_S1_S2_and_S5.pdf]

Table S1. Number of samples, treatment time, and statistical significance thresholds for deregulated genes.

Cell	# Samples	Treatment time	Significance threshold
A549	6	1 min	adj. p. value ≤ 0.05 AND $\vert log_2FC \vert \geq 1$
MCF-7	2	10x30sec (1h interval)	$\vert log_2FC \vert \geq 1$
MCF-7	2	10x30sec (1h interval)	$\vert log_2FC \vert \geq 1$
MCF-7/TamR	2	10x30sec (1h interval)	$\vert log_2FC \vert \geq 1$
MCF-7/TxR	4	10x30sec (1h interval)	$\vert log_2FC \vert \geq 1$
SK-Mel-147	6	3 min	adj. p. value ≤ 0.05 AND $\vert log_2FC \vert \geq 1$
U937	4	2 min	adj. p. value ≤ 0.05 AND $\vert log_2FC \vert \geq 1$
Prostate culture (Gleason 7)	4	3 min	p. value ≤ 0.05 AND $\vert log_2FC \vert \geq 1$

Table S2. Name and description of genes that were found to be significantly deregulated in at least four studies.

	Gene symbol	Description
**1**	***DLX2***	distal-less homeobox 2
**2**	***TSC22D3***	TSC22 domain family member 3
**3**	***EGR3***	early growth response 3
**4**	***MUC20***	mucin 20 cell surface associated
**5**	***FOSB***	FosB proto-oncogene AP-1 transcription factor subunit
**6**	***TINCR***	TINCR ubiquitin domain containing
**7**	***DAPP1***	dual adaptor of phosphotyrosine and 3-phosphoinositides 1
**8**	***HSPA1B***	heat shock protein family A (Hsp70) member 1B
**9**	***IL18***	interleukin 18
**10**	***ISG20***	interferon stimulated exonuclease gene 20
**11**	***JUN***	Jun proto-oncogene AP-1 transcription factor subunit
**12**	***LINC00114***	long intergenic non-protein coding RNA 114
**13**	***ATF3***	activating transcription factor 3
**14**	***PTGER3***	prostaglandin E receptor 3
**15**	***SLC4A5***	solute carrier family 4 member 5
**16**	***ZNF876P***	zinc finger protein 876 pseudogene
**17**	***ZDHHC11***	zinc finger DHHC-type containing 11
**18**	***KLF4***	KLF transcription factor 4
**19**	***ENTPD1***	ectonucleoside triphosphate diphosphohydrolase 1

Table S5. A signature of 34 positively regulated genes based on the meta-analysis of the cohort studies (Fisher's FDR < 0.05).

Gene symbol	Effect Size	effectSize Standard Error	Cochranes Q	Fisher StatUp	Fisher PvalUp	Fisher FDRUp	Description
JUN	2.77	2.248	14.08	62.5	1.20e-09	3.25e-05	Jun Proto-Oncogene, AP-1 Transcription Factor Subunit
NR4A1	1.66	1.061	11.18	60.3	3.17e-09	5.73e-05	Nuclear Receptor Subfamily 4 Group A Member 1
NR4A2	1.51	1.463	10.42	59.0	5.59e-09	7.58e-05	Nuclear Receptor Subfamily 4 Group A Member 2
FOSB	1.97	1.459	6.42	55.2	2.89e-08	2.61e-04	FosB Proto-Oncogene, AP-1 Transcription Factor Subunit
EGR1	3.01	2.115	15.37	54.1	4.70e-08	3.64e-04	Early Growth Response 1
INSIG1	1.94	1.042	6.75	53.1	7.23e-08	4.90e-04	Insulin Induced Gene 1
HMOX1	1.37	0.646	5.94	51.8	1.27e-07	6.24e-04	Heme Oxygenase 1
C11orf96	1.38	0.939	3.71	49.0	4.14e-07	1.72e-03	Chromosome 11 ORF 96
IER5	1.31	1.149	7.15	48.6	4.75e-07	1.72e-03	Immediate Early Response 5
UBC	1.31	0.963	10.07	46.7	1.08e-06	2.65e-03	Ubiquitin C
ARL6IP4	1.33	0.399	3.15	44.6	2.59e-06	4.85e-03	ADP Ribosylation Factor Like GTPase 6 Interacting Protein 4
MYADM	1.56	0.814	9.11	44.4	2.84e-06	5.13e-03	Myeloid Associated Differentiation Marker
HSPA1B	1.37	1.015	8.90	42.6	5.86e-06	7.66e-03	Heat Shock Protein Family A (Hsp70) Member 1B
KLF4	1.01	1.285	6.58	42.5	6.18e-06	7.66e-03	KLF Transcription Factor 4
FOS	1.79	1.845	16.07	40.9	1.16e-05	1.09e-02	Fos Proto-Oncogene, AP-1 Transcription Factor Subunit
RAE1	1.34	0.961	8.51	40.2	1.56e-05	1.39e-02	Ribonucleic Acid Export 1
TNFRSF10D	1.70	0.905	5.21	39.8	1.81e-05	1.55e-02	TNF Receptor Superfamily Member 10d
DNAJB1	1.42	1.375	11.08	38.8	2.72e-05	1.89e-02	DnaJ Heat Shock Protein Family (Hsp40) Member B1
MIR22HG	1.46	1.109	11.92	38.2	3.43e-05	2.05e-02	MIR22 Host Gene
CDKN1A	1.05	1.240	7.87	37.9	4.00e-05	2.27e-02	Cyclin Dependent Kinase Inhibitor 1A
ATP6V0B	1.67	1.474	13.63	37.4	4.76e-05	2.53e-02	ATPase H+ Transporting V0 Subunit B
PTGS2	2.00	1.603	11.14	37.4	4.84e-05	2.54e-02	Prostaglandin-Endoperoxide Synthase 2
PLAUR	1.26	1.068	11.86	36.8	6.18e-05	2.96e-02	Plasminogen Activator, Urokinase Receptor
PRKCB	1.07	0.658	7.17	36.7	6.49e-05	3.08e-02	Protein Kinase C Beta
CSRNP1	1.21	1.371	7.97	36.3	7.59e-05	3.32e-02	Cysteine And Serine Rich Nuclear Protein 1
EMP1	1.21	0.661	6.06	36.2	7.73e-05	3.35e-02	Epithelial Membrane Protein 1
ZNF263	1.45	1.349	13.80	36.2	7.80e-05	3.36e-02	Zinc Finger Protein 263
TIGD1	1.26	0.771	3.24	36.1	8.22e-05	3.41e-02	Tigger Transposable Element Derived 1
BCL6	1.07	0.867	9.15	36.0	8.41e-05	3.42e-02	BCL6 Transcription Repressor
SFN	1.00	0.878	6.53	36.0	8.52e-05	3.42e-02	Stratifin
HLA-E	1.59	1.889	21.96	35.9	8.81e-05	3.49e-02	Major Histocompatibility Complex, Class I, E
RPN1	1.29	0.594	4.48	35.0	1.25e-04	4.23e-02	Ribophorin I
PTGES2	1.02	0.976	5.00	34.7	1.39e-04	4.59e-02	Prostaglandin E Synthase 2
ZNF34	1.08	1.145	7.60	34.6	1.44e-04	4.61e-02	Zinc Finger Protein 34
